# Spatial Simulations of Myxobacterial Development

**DOI:** 10.1371/journal.pcbi.1000686

**Published:** 2010-02-26

**Authors:** Antony B. Holmes, Sara Kalvala, David E. Whitworth

**Affiliations:** 1 MOAC Doctoral Training Centre, University of Warwick, Coventry, United Kingdom; 2 Department of Computer Science, University of Warwick, Coventry, United Kingdom; 3 Institute of Biological, Environmental and Rural Sciences, Aberystwyth University, Ceredigion, United Kingdom; University of Washington, United States of America

## Abstract

Many bacteria exhibit multicellular behaviour, with individuals within a colony coordinating their actions for communal benefit. One example of complex multicellular phenotypes is myxobacterial fruiting body formation, where thousands of cells aggregate into large three-dimensional structures, within which sporulation occurs. Here we describe a novel theoretical model, which uses Monte Carlo dynamics to simulate and explain multicellular development. The model captures multiple behaviours observed during fruiting, including the spontaneous formation of aggregation centres and the formation and dissolution of fruiting bodies. We show that a small number of physical properties in the model is sufficient to explain the most frequently documented population-level behaviours observed during development in *Myxococcus xanthus*.

## Author Summary

Understanding how relatively simple, single cell bacteria can communicate and coordinate their actions is important for explaining how complex multicellular behaviour can emerge without a central controller. Myxobacteria are particularly interesting in this respect because cells undergo multiple phases of coordinated behaviour during their life-cycle. One of the most fascinating and complex phases is the formation of fruiting bodies—large multicellular aggregates of cells formed in response to starvation. In this article we use evidence from the latest experimental data to construct a computational model explaining how cells can form fruiting bodies. Both in our model and in nature, cells move together in dense swarms, which collide to form aggregation centres. In particular, we show that it is possible for aggregates to form spontaneously where previous models require artificially induced aggregates to start the fruiting process.

## Introduction

Bacteria are able to sense their surroundings in order to adapt to environmental change. Most bacteria live in dense populations, therefore other cells constitute a major part of their physical and chemical environment allowing regulatory interactions between cells to be established. The benefits of coordinated behaviour include: more efficient proliferation resulting from a cellular division of labour, access to resources that cannot be utilised by isolated cells, defence against antagonists and population survival by differentiation into distinct cell types [1].

Myxobacteria are Gram-negative, ubiquitous, soil dwelling bacteria that are semi-flexible, and rod-shaped. Cells glide across a surface using the adventurous (A) and the social (S) motility systems [2]. S-motility is coordinated at the leading pole; cells extend type IV pili which can adhere to the surface of other bacteria or polysaccharides, and upon retraction the cell is pulled forward. A-motility is coordinated at the lagging pole; cells are thought to extrude a slime which expands and generates a propulsive force to push cells forward [3],[4]. Myxobacteria display distinct social phenotypes and multicellular behaviours.

*Myxococcus xanthus* is the most commonly studied species of myxobacteria. In response to starvation, cells undergo multiple phases of behaviour culminating in the formation of fruiting bodies and myxospores. The developmental process involves a series of macroscopic changes in colony morphology. A key regulator of development is C-signal ling which occurs when C-signal, a cell surface-associated signal encoded by *csgA*, is exchanged between cells in close contact with one another. C-signal stimulates the expression of *csgA* leading to a rise in C-signal ling throughout development from positive feedback. Different colony morphologies are a consequence of different C-signal ling levels [5]. C-signal ling is thought to affect the reversal frequency of individual cells in a contact-dependent fashion allowing the synchronisation of behaviour [5]–[7].

During vegetative growth cells move in the direction of their long axis, reversing typically once every ten minutes [8]. Under starvation conditions, C-signal accumulates within a cell [5] reducing its reversal frequency [9]. The reduction in the reversal frequency and the effects of A and S motility causes cells to form streams and increases the likelihood of aggregation; cells which cannot reverse tend to remain stuck in one location since their ability to move around obstacles is limited by only being able to move forward [10].

*M. xanthus* cells begin to form fruiting bodies after a prolonged starvation period of approximately 24 h. Starved cells form into large, intricate multicellular aggregates containing between 50,000 and 100,000 cells [11]. The fruiting body is the precursor to sporulation where cells undergo morphogenesis and physically change shape from rods to nearly spherical cells [12]. Inside the nascent fruiting body, a percentage of the cells differentiate into dormant myxospores. This process requires both temporal and spatial coordination in three dimensions, making it one of the most complex and least understood phases of the life-cycle. Relatively little is known about the spatial dynamics of fruiting body construction with research primarily devoted to understanding the signalling mechanisms required to coordinate development rather than the actual physics [13].

There is some disagreement over how fruiting actually begins. O'Connor and Zusman [11],[14] observed that cells appear to orbit around a largely stationary aggregation centre. This led to the *traffic jam* model, which proposes that streams of cells collide together causing the formation of a kernel of stationary cells. Cells move around and over the static centre leading to a mound formation [15]. Work on *Stigmatella* fruiting body formation showed that cells form circular orbits around a base and then move upwards in a spiral fashion around the base, building the stalk on top of it [14],[16]. It was presumed other myxobacteria, including *M. xanthus*, form fruits in a similar way; however, Kuner and Kaiser [17] did not observe the spiralling patterns suggesting that this behaviour is possibly non-essential and may not be intrinsically important to fruiting development. Recent work by Curtis *et al.* [13] suggests that fruiting bodies are formed using a stepped layer building approach; large streams of cells forming sheets collide causing a rapid build up in density at the meeting point. Cells in one of the opposing streams are forced upwards and over the other, similar to tectonic plate movements. The displaced cells are supported on top of the dense layer of cells and extra-cellular polysaccharide (EPS) underneath and begin to spread out forming a new layer. As the new layer becomes more dense itself, cells at the centre start to get pushed upwards to form a new layer and the process repeats causing the formation of an expanding mound of cells.

Previous computational models of fruiting body development [18]–[20] are based on the orbiting *traffic jam* model and rely upon the artificial induction of an aggregation centre to start fruiting body development, typically by making a subset of cells stationary. In this paper, we take a different approach and use an off-lattice Monte Carlo simulation to show how cells can spontaneously aggregate to form layers and fruiting bodies based on the observations of Curtis *et al.* [13]. The motivation of this work is to gain an increased understanding of fruiting, by examining the physical properties driving cells to engage in fruiting, using mathematical and computational modelling.

## Model

To study fruiting body development in the model myxobacterium *M. xanthus*, a Monte Carlo model of the fruiting body dynamics was developed. Similar stochastic models have been used to study three-dimensional fruiting formation in *Dictyostelium discoideum* [21] so some of those concepts were adapted to derive the model of myxobacteria fruiting.

### Boundary conditions

Periodic boundary conditions were disabled in the 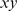-plane since it does not make sense for cells to be able to push through the floor nor move through the ceiling for which there is no physical interpretation. Boundaries are maintained with a boundary energy term which severely penalises a cell for attempting to cross a particular domain boundary. The energy penalty is several orders of magnitude larger than the value any of the other energy terms might produce so it is nearly impossible for a configuration with these domain crossings to be favourable.

### Cell influx

Fruiting body formation requires a highly dense region of cells to seed aggregation. To achieve such a density at the start of simulation would require cells to be placed so that they fill all available space on the floor of the simulation volume. Even under these conditions, the cell density is usually not sufficient to seed fruiting, and the lack of space for movement would inhibit cell motility. Biologically, fruiting bodies are thought to form from the confluence of streams of cells resulting in the cell density increasing over time [4],[22]. To capture this behaviour in the simulations, *entry zones* were placed around the edges of the simulation volume (see Figure S3). Entry zones allow new cells to be introduced into the simulation over time to model cell influx. A maximum influx rate (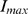) can be specified to govern how quickly new cells enter the simulation volume. The actual influx rate is stochastic and less than the maximum influx rate, 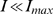, and is determined by the amount of free space within the entry zones where new cells can be placed. New cells are placed at random locations by periodically sampling the entry zones to see if there is free space to place a cell and then placing a cell if the maximum influx rate (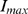) has not been exceeded. Fruiting requires a high cell density and simulating a finite number of cells makes it problematic to assemble enough cells in an area to form a fruit; the cell density is never high enough. A finite number of cells may clump and partially aggregate but they are unlikely to form a fruiting body. With the entry zone model, a constant cell density can be maintained to sustain fruiting body growth.

### C-signalling

Cell reversals are thought be controlled by C-signal stimulating the complex Frz pathway, however the exact function of each component has yet to be determined [23]. We therefore model the macroscopic behaviour of the pathway, where an internal phase switch is used as an abstract representation of C-signal. The switch increments until it reaches 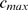 at which point it resets and the cell reverses. The switch can be perturbed by signalling between neighbouring cells to make reversals happen more quickly, by a factor proportional to the number of collisions a cell experiences with its neighbouring cells. The function is therefore:
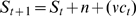
(1)

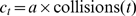
(2)where 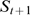 is the new cumulative value, 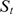 is the current value, 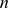 is a basal increase factor, 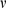 is the signal strength, and 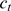 is the level of C-signal ling a cell experiences at time 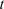, defined by the collisions a cell experiences with each of its neighbours and 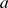 a collision factor.

In this work we keep the model of C-signal ling quite simple, as our goal is to explore other factors which can facilitate the formation of aggregates and fruiting bodies. Experiments indicate that even 15 hours into starvation, levels of C-signalling are sufficient to reduce the rate of reversals to once every 22 minutes [24]. Moreover, these experiments show that the slowdown in reversal induces a 15-fold increase in travel distance, in what could be considered a ‘unidirectional behaviour’. We approximate this low frequency of reversals by considering cells which have come near to an aggregation as non-reversing, reflecting the approach taken in other simulations [18],[19]. Nevertheless, in simulations of fruiting C-signal ling levels and collisions are monitored, enabling the imposition of a threshold C-signal ling level governing the induction of sporulation.

### Implementation

**Figure 1:**
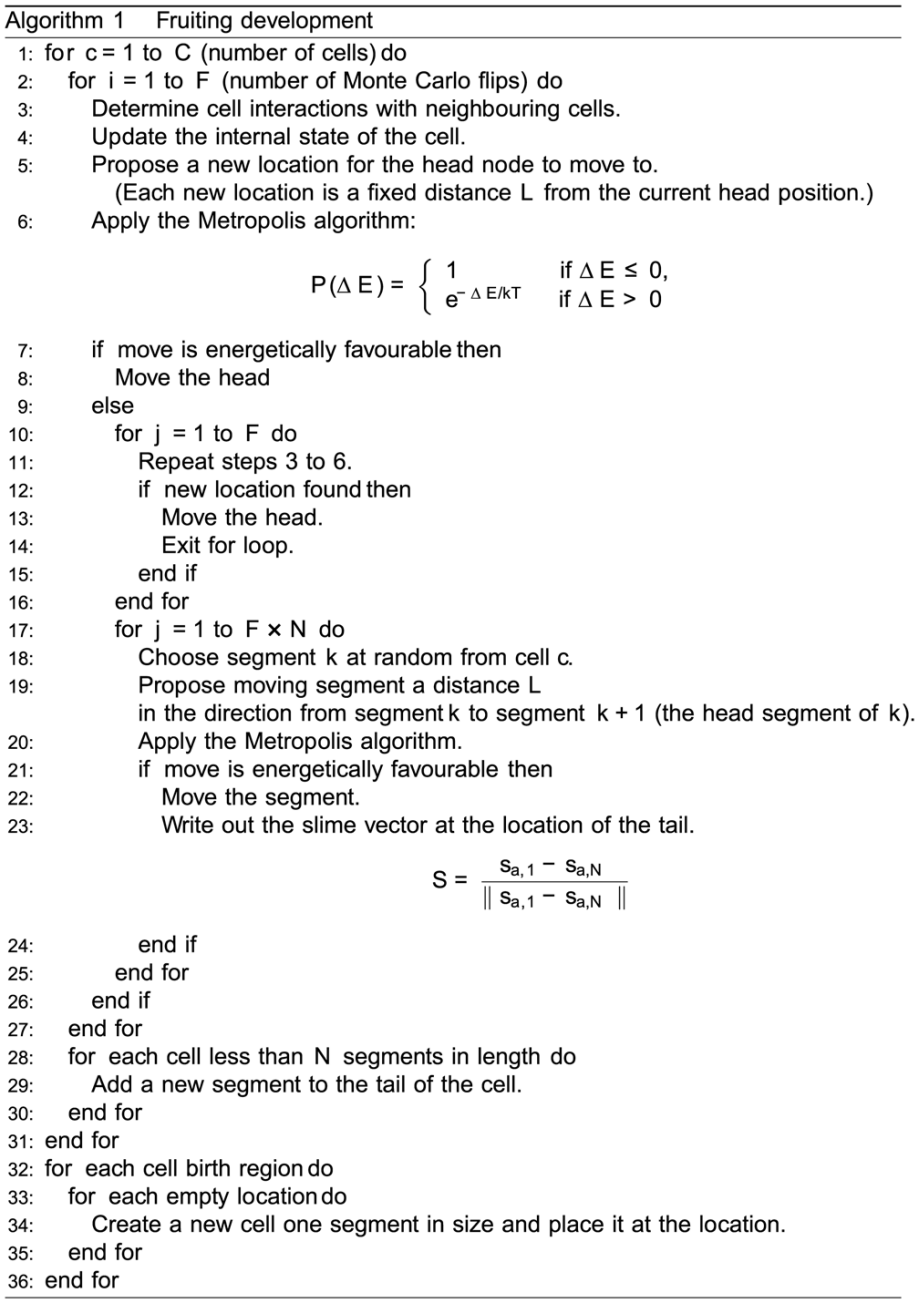
Algorithm driving the simulation of fruiting development based on the Metropolis algorithm.

Figure 1 describes the program used for simulation. The Metropolis algorithm [25] is used to determine the acceptance probability of making any particular change. Simulations were carried out using a volume equivalent to 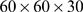 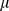
m.

The model captures the physical dynamics of the cells using the method proposed by Glazier and Graner [26]. A Cellular Potts Model is a probabilistic Cellular Automata with Monte-Carlo updating, where the next state of the lattice is chosen by evaluating a *Hamiltonian* equation used to calculate the probability of accepting lattice updates. The original Potts model [27] was developed to capture behaviour at the level of statistical mechanics but has been successfully generalized for a variety of domains.

**Figure 2:**
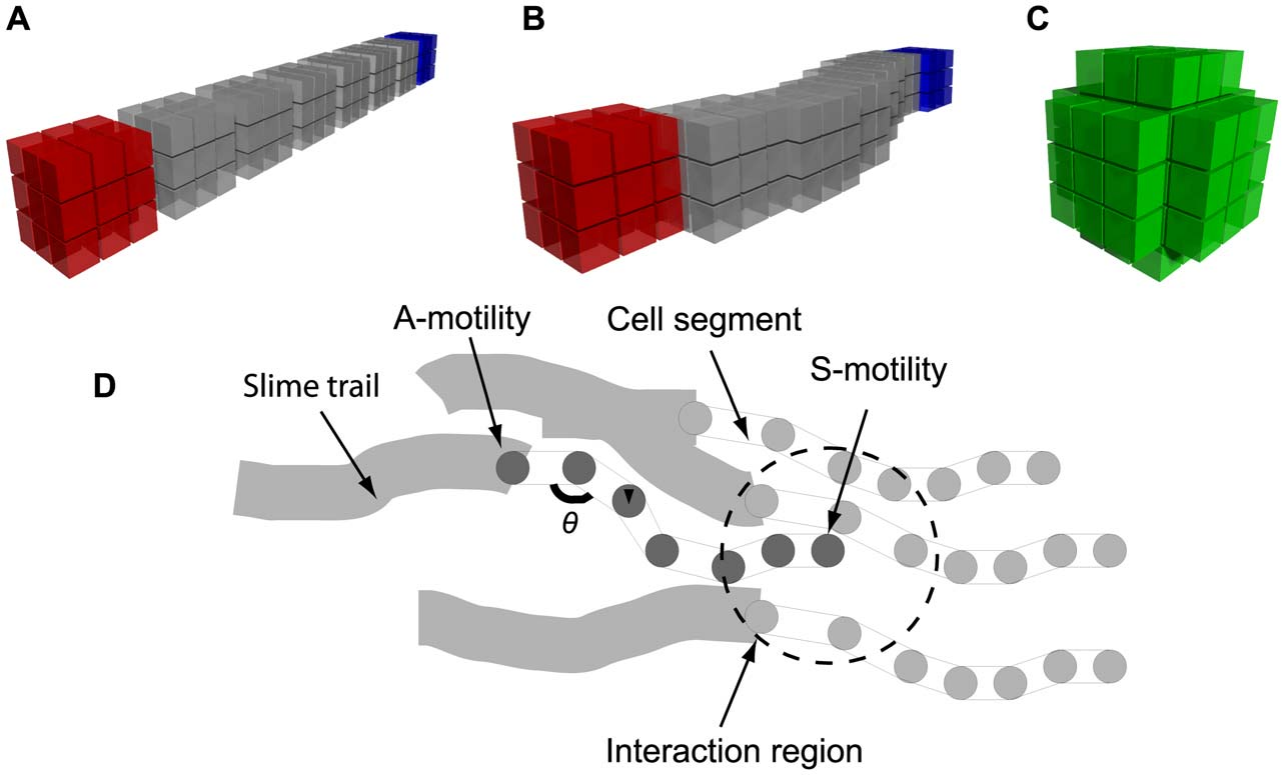
The physical characteristics of model *M. xanthus* cells. (A) Fruiting body cells have eight connected segments: a head (red), a tail (blue) and six body segments. Each segment comprises 27 segment nodes in a cube formation. (B) Segments move independently allowing the cell body to be flexible. Overlap between segments allows the cell to maintain a continuous cell volume. (C) During sporulation cells change shape becoming immobile single segment spores. A spore is represented as a three-dimensional sphere of segment nodes. (D) Cells are semi-flexible and move using the effects of the A and S motility systems. The head segment uses an interaction region to determine the neighbouring cells it can interact with.

The tuning of a Cellular Potts Model is based on finding an appropriate Hamiltonian function and appropriate parameters for this function. The heart of our model is the development of a set of terms that correctly describes important physical characteristics of the *M. xanthus* cell (see Figure 2). The level of detail used needs to be balanced with the computational cost of these calculations.

The following Hamiltonian function, inspired by the approach of Izaguirre *et al.* [28], describes the energy components of *M. xanthus* we use:
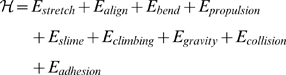
(3)


A separate collision resolution algorithm such as used by Wu *et al.* [29] was not required since collision avoidance is a feature of the Hamiltonian.

In the following presentation of each of the components of the Hamiltonian, we use boldface fonts to indicate vectors, and the cap operation  to denote an average or mean vector.

### Stretching energy

*M. xanthus* cells are modelled as having a finite volume and stable shape; cells can be squashed to an extent but they maintain a rod shaped structure except during sporulation. Cell length governs a cell's length and is analogous to the spring constant in Hooke's Law.
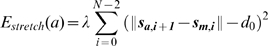
(4)where 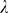 is a dimensionless stretching coefficient, 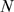 is the number of segments in cell 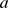, 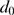 is the optimal distance between segments, 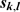 is the vector position of segment 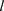 in cell 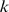 and 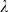 a dimensionless stretching coefficient. Stretching energy is defined as a squared sum which compares the distance between the centres of neighbouring segments 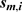 and 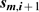 to 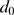 and penalises a cell for allowing segments to get either too close or too far apart.

### Alignment energy

In close proximity, cells tend to align with each other reflecting the effect of the S-motility engine. Cells extend Type IV pili from their leading pole which grab onto neighbouring cells. Upon retraction this pulls a cell closer to the neighbour it latched onto [4]. The natural consequence of this movement is the alignment of cells [30].
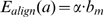
(5)

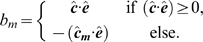
(6)

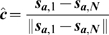
(7)

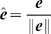
(8)

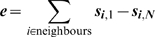
(9)where 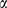 is a dimensionless alignment coefficient, 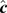 is the normalised average direction of the cell, 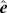 is the average direction of all the cells in a local neighbourhood surrounding cell 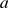. 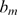 reflects that cells tend to turn through the acute angle to align with other cells in either direction.

### Bending energy

Each cell in the model has a semi-flexible body which must maintain a certain stiffness, otherwise the cell would fold up upon itself. Incorporating bending energy in the Hamiltonian ensures that the radius of curvature of a cell does not exceed a threshold causing the cell to flail uncontrollably and unnaturally.
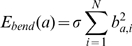
(10)

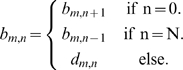
(11)

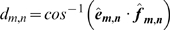
(12)

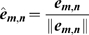
(13)

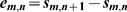
(14)

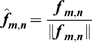
(15)

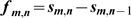
(16)where 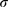 is a dimensionless bending coefficient, 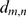 returns the angle between 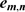 and 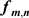, 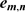 is the average direction of segment 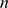 of cell 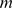 and 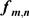 is the vector between the segment ahead of 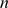 (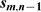) and the segment behind (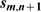).

### Propulsion energy

The A-motility system provides myxobacteria cells with propulsion. Cells extrude a polysaccharide slime from nozzles at their lagging pole, which is thought to expand when hydrolysed and push a cell forward [4]. This effect is modelled using a propulsion term which causes cells to move preferentially in the average direction of the cell simulating the slime pushing a cell along.
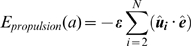
(17)

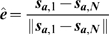
(18)

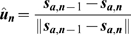
(19)where 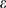 is a dimensionless propulsion coefficient, 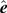 is the normalised average direction of the cell and 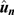 is the update direction of segment 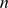 of cell 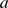. Each segment moves towards where its head segment was previously, unless this causes segments to become unaligned.

### Slime trail following energy

As well as extruding slime to move, cells can also detect slime trails left by other cells and preferentially follow them. This allows cells to follow other adventurous cells and leads to the formation of streams that can break away from the main colony. Slime following is complementary to A-motility. As each cell moves, it deposits a slime trail. Early evidence of the presence and effect of slime trail following is provided by the videos created by Reichenbach [31].

This effect of slime following is represented in our model as a set of normalised vectors representing the average direction of a cell. The slime ages over time and is eventually removed. Cells can sense slime trails within a limited neighbourhood around them. Using a weighted sum of the all slime trail directions based upon their age, the average slime direction is calculated and cells preferentially follow that. We use a weighted sum to account for the fact that a cell is more likely to follow a large slime trail than a small one.
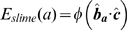
(20)

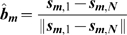
(21)

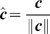
(22)

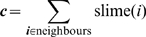
(23)

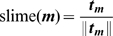
(24)where 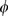 is a dimensionless slime following coefficient, 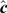 is average direction of the slime trails in a neighbourhood and 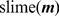 is the normalised direction vector of the slime trail at location 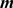.

### Climbing energy

Curtis *et al.* [13] observe that cells appear to move in sheets towards each other and, upon impact during a collision, cells from one sheet can move up and on top of the other. This is consistent with O'Connor and Zusman [11] who suggest that cells appear to behave as independent sheets. This effect has been modelled so that it is somewhat analogous to a snow plow, which is pushed forwards into the snow pushing the snow up and away. In a similar way it is proposed that the oncoming force of a sheet of cells is sufficient to push oncoming cells up and direct them over and on top. Each cell monitors the number of head-on collisions it has, and the more the collisions the greater the chance of it being pushed up. Cells are not forced to always be pushed upwards, as this would be imposing an artificial constraint on the system, instead cells prefer regions of lower cell density where they are freer to move. Some cells will be pushed outwards away from the stream, but the majority will be pushed upwards since this is the only region of free space available.

**Figure 3:**
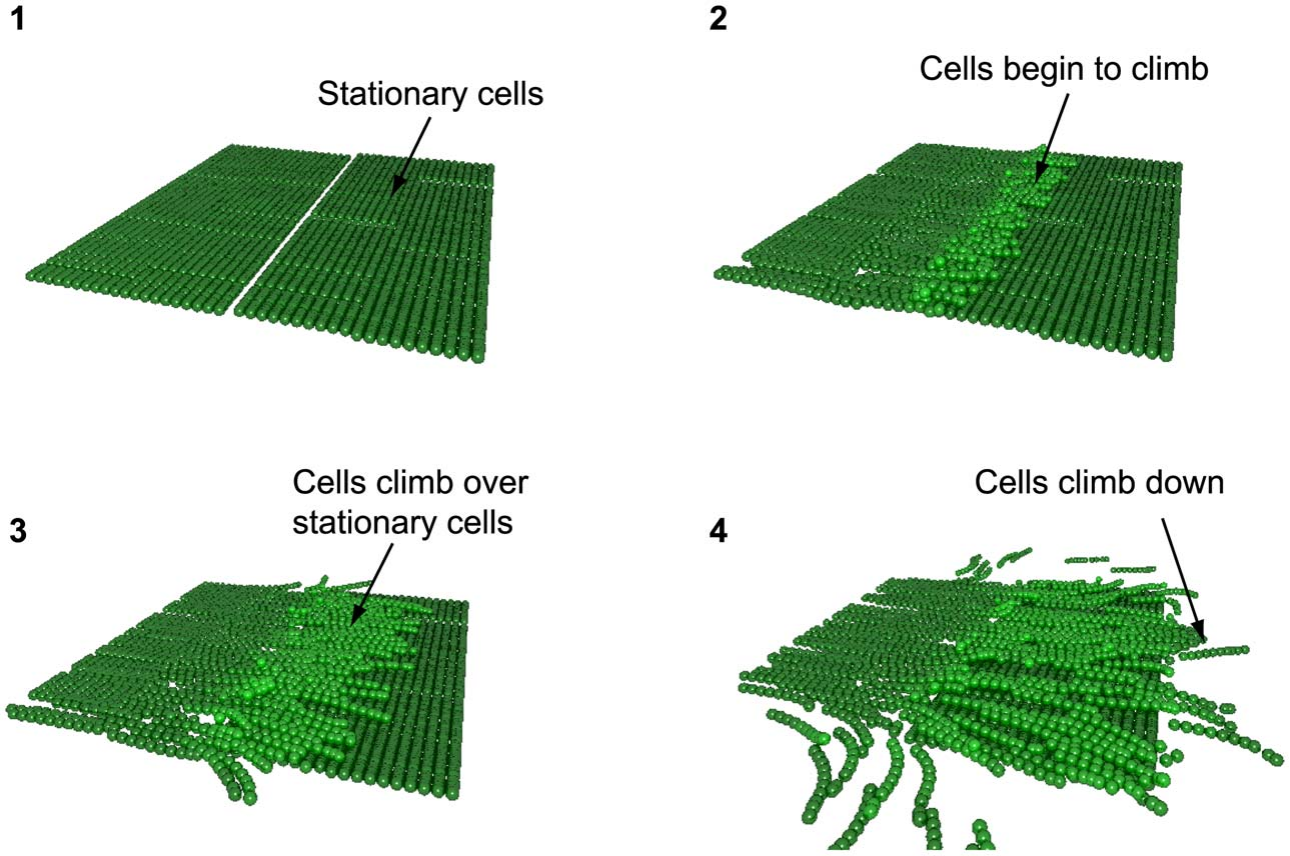
A model of cell climbing and layer formation. When cells encounter an obstacle such as stationary cells they either attempt to move around it or climb over it.

Curtis *et al.* [13] propose that when two sheets of oncoming cells encounter each other, individual cells have a proclivity to move out of the potential “traffic jam” that can ensue and typically this is upwards so one sheet of cells effectively moves over the other. A simulation of climbing cells which form layers can be seen in Figure 3. The energy term we use, described below, encourages cells to move upwards, proportionally to the number of oncoming cells they interact with.
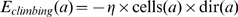
(25)

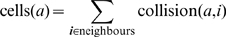
(26)

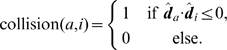
(27)

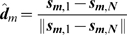
(28)

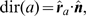
(29)where 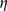 is a dimensionless climbing coefficient, 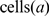 determines the number of oncoming cells, 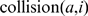 determines if two cells are moving in opposing directions by examining the dot-product between the normalised average direction (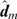) of each pair of interacting cells, and 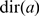 compares the direction cell 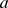 tries to move in (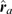) with a normal vector (typically a normal to the 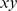-plane).

### Gravitational energy

In a three-dimensional model, cell movement in the 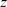-axis needs to be controlled so that cells do not randomly climb into empty space and defy gravity. The other energy terms do not prevent cells from climbing so gravity is therefore introduced as an energy penalty for trying to climb; the steeper the climb the greater the penalty. An object acting under gravity requires the greatest amount of energy to directly oppose the force and move in the opposite direction (upwards). It should be noted that the use of the dot-product means there is no net effect of this term for a cell moving horizontally in the 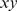-plane; since gravity is a constant, there is no change in energy from moving between two positions with a direction vector perpendicular to the direction of the gravitational force.
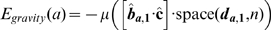
(30)

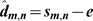
(31)where 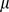 is a sensitivity parameter, 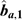 is the normalised update direction of the head segment, 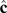 a normalised direction vector pointing towards the ground, 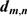 is a location below the centre of segment 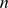 of cell 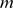 and 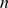 is a local neighbourhood surrounding 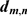.

### Collision energy

In order to capture natural elasticity and bending, each cell is modelled as a number of segments each with a finite volume. Segments must exert a repulsive force between themselves to prevent cells colliding. This force contributes to the Hamiltonian as follows.
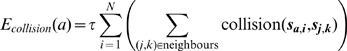
(32)

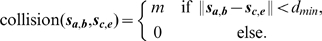
(33)where 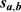 is the position of segment 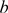 of cell 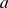 and 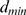 is the minimum distance allowed between segments of difference cells. The collision energy compares the distance between a segment and the neighbouring segments 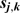 around it and severely penalises a cell for getting too close to another. Although the centres of segments cannot occupy the same space, a small overlap is allowed to model deformation effects of cells in close proximity. This is required because of the rigid segment shape which would otherwise not allow for this type of effect.

### Adhesion energy

Extracellular polysaccharide (EPS) secreted by the cells during aggregation formation appears to play an important role in the formation of the physical structure of the fruiting body [11],[17]. The exact role of the slime has yet to be elucidated due to the methods used to collect data and the very high cell densities within the fruit, making it difficult to resolve individual cells. Electron microscopy can resolve cells at higher resolutions [11],[16] but this can only take a snapshot of a dynamic process and is unsuitable for tracking cells over a relatively long time period.

In a dense region, cells generate a lot of EPS with a fruit being a large amalgamation of cells within an EPS matrix. The EPS is likely to exert a surface tension effect causing cells to stick together rather than drifting apart. This is separate from the slime trail following effect as it is non-directional, acting over the whole cell area. If two cells are close to each other and encased in slime, breaking them apart requires extra energy to counter the adhesive effects of the slime. In contrast to the climbing effect, here cells experience an energy penalty for breaking apart. It is a form of non-specific attraction and operates over short ranges since two cells several cell lengths apart will not affect each other; only cells in close proximity experience adhesion.

The high density of cells in a swarm and fruiting body means there is a large amount of polysaccharide slime produced which encases all of the cells in a slime matrix [11],[16],[32]. The slime casing prevents cells coming apart, for example even with a rotary shaker. This matrix effects an adhesive force on the cells making it harder for cells to move apart from each other. Cells typically aggregate at a colony edge due to surface tension effects making it difficult to escape the colony [20]. This effect is different from the effects of A-motility and is a global property of a large mass of cells.
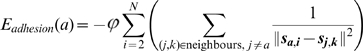
(34)where 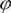 is a dimensionless adhesion coefficient, 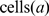 determines the number of oncoming cells, 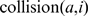 determines if two cells are moving in opposing directions by examining the dot product between the normalised average direction (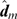) of each pair of interacting cells, and 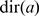 compares the direction cell 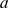 wants to move in (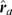) with a normal vector (typically a normal to the 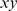-plane).

### Sporulation

As a fruiting body matures, 65–90% of cells lyse, with the remaining cells going on to form myxospores [33],[34]. Spores appear to migrate to the centre of the fruiting body with motile cells remaining on the outside and the periphery [11].

The fruiting model was extended to incorporate sporulation and its effects on fruit formation. Each cell is given a type: *motile* or *spore*. Motile cells accumulate C-signal from collisions with other motile cells. Once C-signal exceeds a threshold (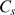), cells convert to non-motile spores. Spores can be moved by motile cells pushing them. Each cell type has its own Hamiltonian governing its behaviour. Normal cells continue to use the Hamiltonian defined in Equation 3:
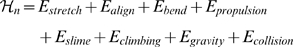
(35)


However spores are non-motile cells with a fixed size and shape, and the Hamiltonian controlling them loses terms associated with autonomous cell motion and is therefore simpler:
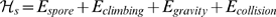
(36)


Although spore cells are immobile, other motile cells can move them during collisions when they collide and through adhesive effects between cells.
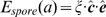
(37)

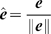
(38)

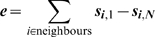
(39)where 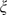 is a dimensionless coefficient, 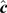 is the normalised average direction of the cell, 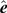 is the average direction of all the cells in a local neighbourhood surrounding cell 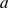. The term 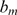 reflects the tendency of cells to turn through an acute angle to align with other cells in either direction.

### Simulation parameters

The parameters used in the simulation are listed in Table 1; the same parameters were used in our previous model of rippling behaviour [35]. Some of these parameters reflect the level of abstraction that approximate the level of behaviour observed through video microscopy. The 7∶1 length to width ratio reflects evidence from [36]. The volume of each cell was set so that thousands could be fit into a volume large enough to hold a fruiting body without requiring unreasonable amounts of computational memory. Representing cells via eight segments seemed to provide a reasonable approximation of the degree of flexibility observed in various phases of the lifecycle.

**Table 1:** Parameters for models used to simulate fruiting body formation in *Myxococcus xanthus*.

Name	Value	Description
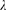	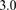	stretching energy parameter.
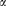	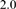	volume energy parameter.
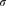	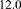	bending energy parameter.
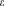	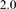	propulsion energy parameter.
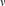	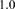	climbing energy parameter.
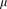	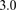	gravity energy parameter.
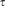	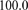	collision energy parameter.
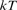	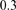	Boltzmann constant 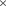 temperature.
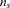	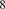	Number of segments per cell.
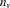	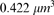	Segment volume (number of segment nodes).
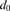	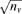	target distance between adjacent segments.
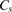	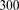	C-signal sporulation threshold.

Motility parameters were based initially on experimental evidence [37],[38] to get an idea of the speed of cells, and then tuned so that cells moved at the correct speed given their size and volume in the simulation environment. Likewise, parameters governing the flexibility of cells were based initially on [39]; other parameters were tuned relative to these to emulate the cell motion patterns observed in nature.

## Results

### Model of fruiting body formation

Simulations were carried out in a three-dimensional environment using a model based on our previous stochastic model of myxobacteria motility [35]. *M. xanthus* is approximately 5–7 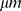 long and 0.5 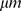 in diameter so the model cells were given a length to width ratio of 7∶1. Each cell was composed of eight three-dimensional segments (see Figure 2) with each segment being composed of 27 segment nodes arranged in a cube formation. Segments were allowed to overlap so that cells maintained a continuous volume and the correct aspect ratio despite being made of multiple separate segments (see Figure 2 b). The physical behaviour of cells was described using a Hamiltonian function whilst the internal state was described using ordinary differential equations (ODEs).

### Cell adhesion

The EPS surrounding cells is rarely considered in models; however, in our model we found that slime can have an essential role in fruit formation. The Hamiltonian includes an adhesion term, which generates energy proportional to the inverse square of the distance between any two cells in a neighbourhood. It is more energetically favourable for cells to remain close to other cells otherwise there is a severe penalty for moving apart that increases exponentially with distance. An inverse relationship was chosen so that long range interactions are weak; cells towards the perimeter of the local neighbourhood should not exert the same influence as cells in close proximity. Adhesion acts to control the viscosity of the slime determining how easy it is for cells to move through it. The amount of slime and thickness varies depending on the stage of fruiting and the cell density [16]. We note that Curtis *et al.* reported that a *pilA* mutant produces less EPS and this inhibits fruiting body formation [13]. It is not known biologically whether the effect of the *pilA* mutation is a consequence of reduced EPS production, or due to altered motility properties. Therefore, a direct comparison with the *pilA* mutant described by Curtis *et al.* is not possible.

Figure S1 shows the effect of varying the strength of adhesion on a stream of cells. When there is no adhesion, cells at the front of the stream are able to move adventurously, causing the stream to break down into a number of smaller streams which diverge. As the adhesion strength (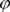) is increased, cells remain much closer. When 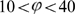 cells tend to stay as one or possibly two large coherent streams. When 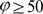, the slime is so viscous that cells are no longer able to move.

### Aggregate formation

**Figure 4:**
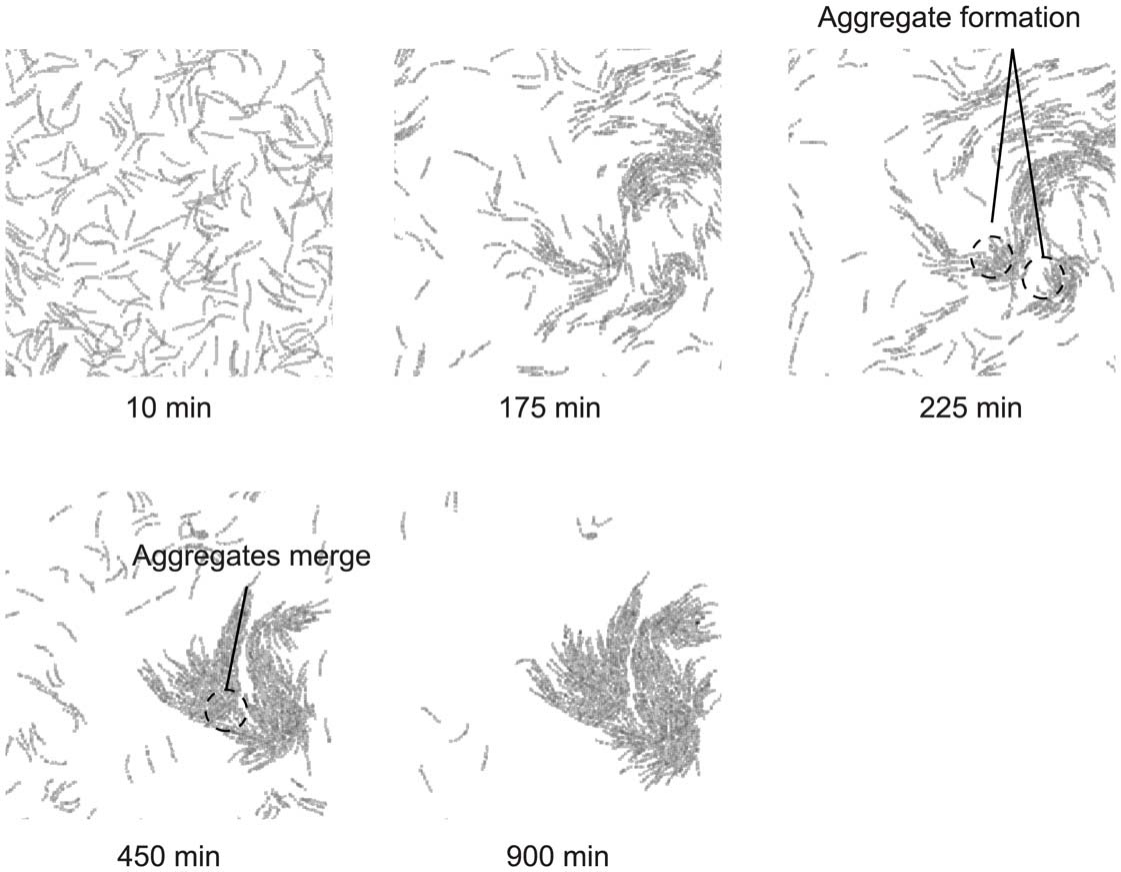
Simulation of cell aggregation. 300 cells were randomly oriented and allowed to interact. Two aggregation centres spontaneously form. They eventually merge into one large aggregation centre where a fruiting body can form. In this model a fruit will not form as the cell density is not great enough to sustain building.

Fruiting begins with streaming and the confluence of streams to form aggregation centres. The fruiting model presented here allows cells to spontaneously form streams and aggregation centres (see Figure 4). A simulation consisting of 300 cells was run three times to determine the efficacy of streaming and aggregation (model parameters are given in Table 1). Cells were initially randomly distributed. After approximately 100 min of simulated real time, cells formed into streams regardless of their initial configuration. Cells aligned and formed small streams which joined other streams when they came into contact. After 300 min cells typically formed an aggregate, which expanded as the the majority of cells joined it.

The effects of motility along with cell adhesion causes model cells to form streams. As the streams approach an aggregation centre, cells will attempt to avoid collision and alter course. They begin to move around the aggregate causing the stream to change direction and form the characteristic spiral patterns observed by O'Connor and Zusman [11].

### A finite number of cells prevents fruiting

In a model with a finite number of cells, it is difficult to achieve a high enough cell density to maintain aggregates. There is an upper bound on the density of cells in a mono-layer above which cells will not have enough space to move and be able to engage in activity. With a high cell density which still allows cells to move, it is possible to get aggregation, but once the fruit starts to form, the number of cells moving into the aggregate will not be sustainable and the fruit will simply dissociate. This type of model is also unrealistic because in reality, an aggregate would be surrounded by other cells and not sit in isolation as more cells join it. Although it would be ideal to model a vast mono-layer of cells to ensure there were a sufficient number of cells to a form a fruit, computational limitations (typically memory) restrict the size of a simulation.

Figure S4 shows the output of a fruiting body simulation using a finite number of cells. 1600 cells were arranged into two opposing sets of streams with one set perpendicular to the other. The streams move into each other and collide. In the aggregation centre, some cells push upwards and move over others forming new layers and the base of a stalk. The effect of using a finite number of cells becomes apparent after 300 time steps when the stalk begins to disassociate. The cells organise themselves into a stack four layers thick, but since there are no more cells to expand the base layer, the upper cells begin to climb down and move away from the fruit. Once a few cells move away, a mass exodus is triggered causing all of the cells to move away. The formation and subsequent rapid dispersion of fruits will occur at any point where an aggregate forms. This effect will be more apparent on subsequent aggregation formations since the number of cells within the mass is unlikely to be as high as in the initial formation so the deterioration will be more pronounced.

### Fruit dispersal

Curtis *et al.* [13] observed that during the initial stages of fruiting, small fruiting bodies would sometimes repeatedly start to form and then dissipate before a stable fruiting body finally formed (see Figure 1 in [13]).

The formation of transitory aggregates can be explained by adjusting the cell influx rate. The simulations maintained the same initial conditions as the previous fruiting simulation, except the rate of influx was altered. Figure S2 shows a snapshot of a simulation where the influx rate was reduced by 90%. Although a fruiting body begins to form it rapidly dissociates over time. Cells accumulate and the stack expands outwards from the centre for approximately 200 min after which the fruit collapses and the cells begin to disperse. The cell density remains too low for cells to attempt a new fruit formation suggesting that influx could be a primary driving factor behind development.

**Figure 5:**
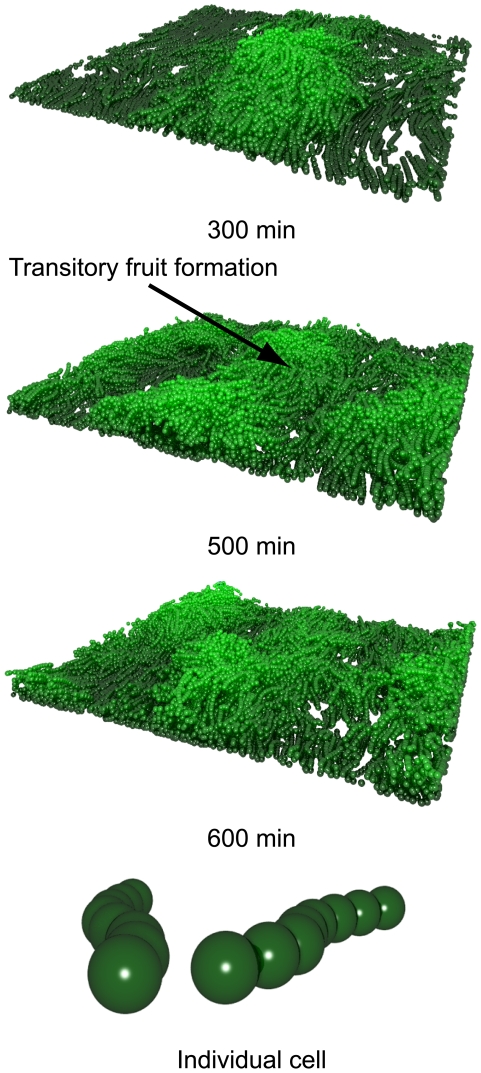
Three-dimensional plots showing the fruiting body formations can spontaneously form and re-form. Mound formations are coloured by height from 0 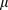
m (dark green) to 30 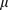
m (light green).

The base influx rate was selected to ensure a constantly high cell density to enable fruit formation. Lower influx rates promote transitory fruiting body formation and dissociation. Figure 5 shows three-dimensional snapshots of fruiting development when the influx rate was reduced to 25% of the base value. After 500 min, three small mounds have formed; however, they dissociate and new mounds form. This agrees with experimental evidence showing transitory aggregates [13]. If the simulation volume were enlarged by several orders of magnitude (which has not been computationally feasible), we predict that as fruiting bodies disperse, a cohesive layer of cells would form and drift off. This would meet other disparate layers from other dispersed fruits and further fruiting development would be initiated where they collide. The process would repeat leading to multiple transitory fruit formations [13]. The prerequisite for this to occur is a sufficiently high cell influx that allows a fruit to form but at a sub-optimal rate such that development cannot be sustained. The fruiting body must be sufficiently large so that, when cells leave it, they form a layer of equal density to the initial layers so that fruiting can occur spontaneously at other locations. The influx rate appears to be the rate limiting step in controlling fruiting growth; there is a point where the number of cells forming new layers will begin to exceed the number of cells flowing into the system so the development of new layers is arrested.

The influx rate we use here refers specifically to the addition of new cells into the simulation volume. The effect of EPS adhesion also affects influx rate into a local area, since it governs cell alignment and density. The *pilA* mutant described by Curtis et al [13] can be approximated through the EPS model described here.

### Sporulation stabilises fruiting bodies

**Figure 6:**
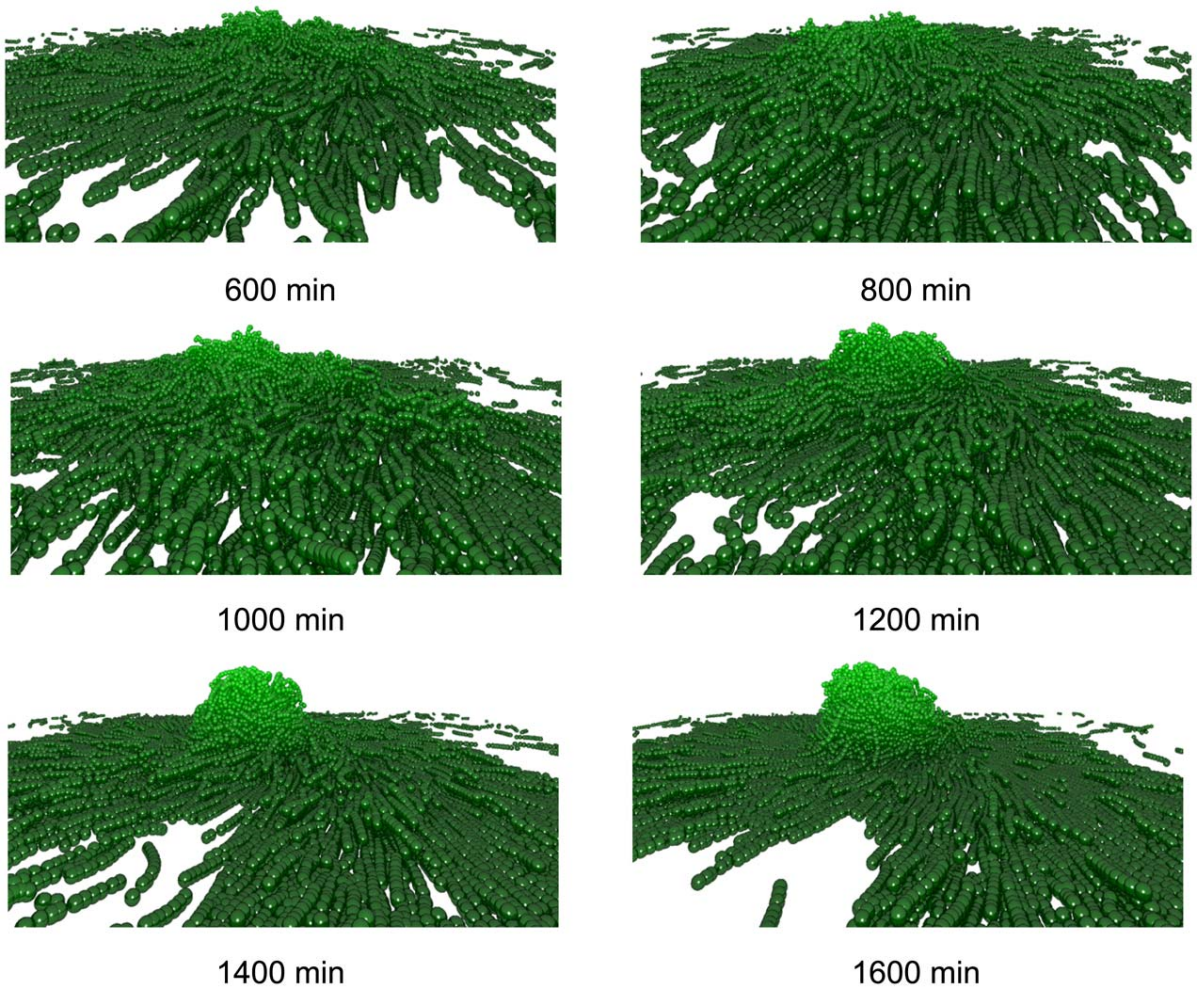
Three-dimensional view of fruiting body simulation when cells are allowed to sporulate. After 24 h a single, stable fruit has formed that continues to expand outwards and upwards forming a hemispherical mound.

Figure 6 shows how mound formation in the fruiting simulation varies over time. The motile cell count rises sharply during the first 16 h of simulated real time as cells accumulate within the fruit and surrounding area. After this time point, a combination of the cell density and the aggregate size makes it more difficult for new cells to enter the aggregate. The high cell density ensures constant C-signal ling triggering a constant rise in myxospores, which occupy an increasing fraction of the aggregate. Fruiting development begins after 10 h with a small mound formation. This rapidly expands and develops into a fruiting body after 24 h around which motile cells orbit in stream formations. Towards the periphery of the fruit, the cell density rapidly decreases leaving only a thin layer of cells (less than three cells deep) in the regions not occupied by the fruit.

**Figure 7:**
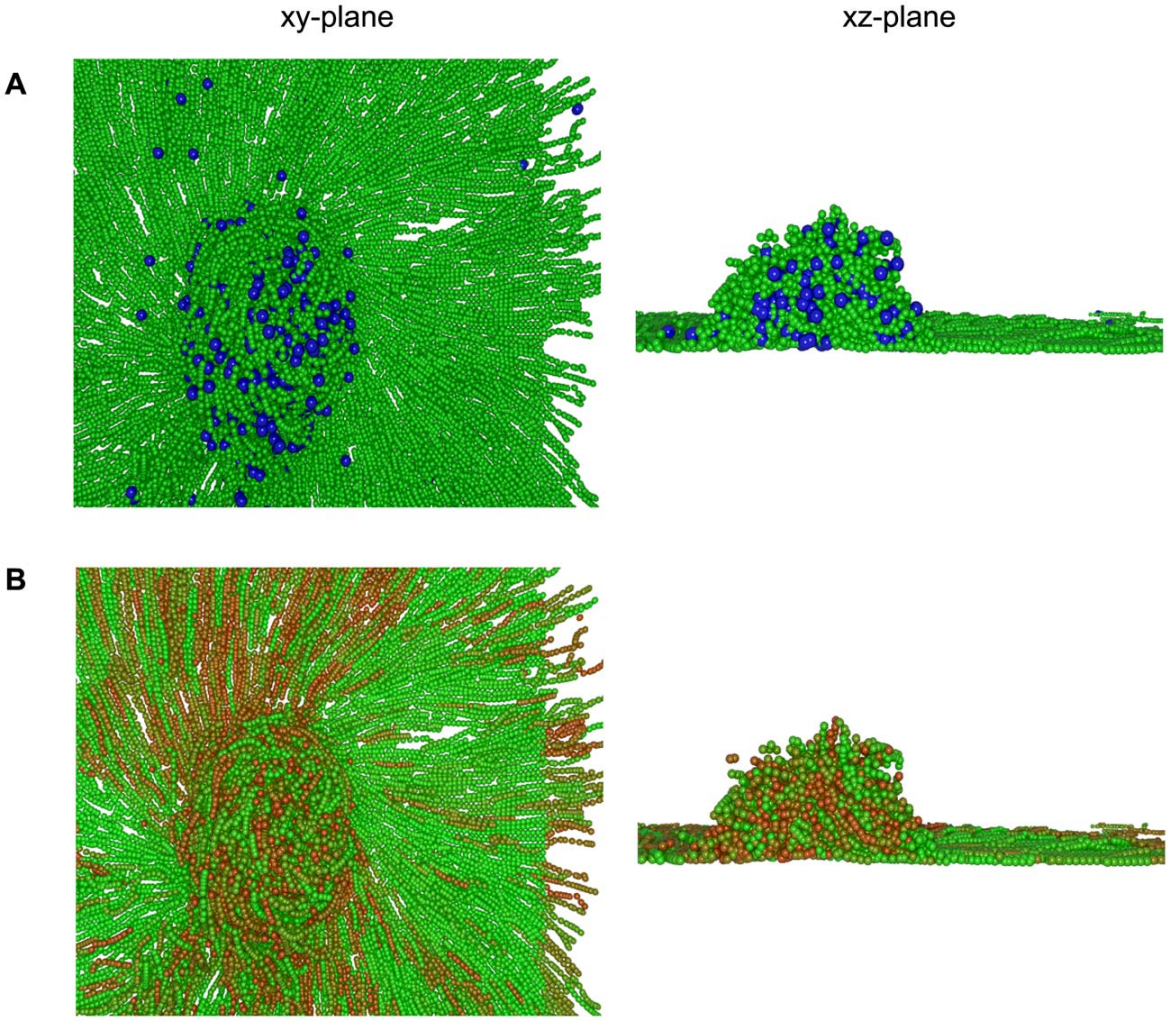
The localisation of C-signal and myxospores within a fruiting body. Simulation is shown after 24 h. (A) View of the simulation showing motile cells (green) and myxospores (blue spheres). (B) View of the simulation showing the accumulated C-signal in each cell. C-signal is a dimensionless quantity measured between zero (green) and 400 units (red).

C-signal is not evenly distributed throughout the colony. Cells within the fruit collide much more frequently with other cells so they accumulate C-signal faster (see Figure 7 a) and sporulate faster (see Figure 7 b). The majority of C-signal ling occurs within the fruit hence spores are formed within or close to the fruit centre and are pushed into the centre by the motile cells.

The large hemispherical aggregate agrees with the formations observed by Kuner and Kaiser [17]. The aggregate is stable due to the spores that occupy the centre of the mound (see Figure 7 a). The spores limit the movement of the motile cells causing them to stall more frequently in the aggregate and expanding the size of the traffic jams. The motile cells push the non-motile cells towards the centre of the aggregate in agreement with existing data [11]. Cells are highly crowded and aligned, forming streams and sheets. This is in agreement with the observations of O'Connor and Zusman (see Fig. 6A in [11]). Video S1 is a video of simulation output showing the formation of the fruiting body.

## Discussion

A precise understanding of how and why myxobacteria cells form fruiting bodies remains elusive; however, we can start to address the issue of fruiting body development from a theoretical perspective and provide a possible explanation of how they form. The models presented here indicate that fruit formation can be simply a natural consequence of cell behaviour without any form of centralised coordination. The lack of reversals makes cells prone to collisions [10]. Aggregation centres tend to form quickly as small streams of cells frequently collide and block each other's path. Without the ability to reverse, cells are forced to remain stuck in their current position. Any cells that join the tail of the trail become stuck as well leading to a traffic jam and the formation of an aggregate. It should be noted that these small streams are typically too small to trigger cells to instigate climbing since although the cells are blocked, the density of neighbouring cells is not generally sufficient to support a new layer of cells on top of it.

A primary goal of the modelling work presented here is to show that a physical and biochemical model can explain multiple phases of behaviour. While it is beyond the scope of this work to fully address *all* factors controlling the myxobacteria life-cycle, we previously showed that a similar Monte Carlo model of cell physics can explain rippling and the behaviour of cells in the early stages of starvation [35]. We also modelled a C-signal ling mutant by switching off the C-signal ling component in the Hamiltonian and observed vegetative behaviour in agreement with Curtis *et al.* [13]. By adjusting the C-signal ling levels of the model presented in this article, cells can be shown to revert to responding to C-signal with a higher incidence of reversal.

Cell climbing was an important factor in governing how well aggregates formed in our model. Cells were allowed to climb at any angle, but it was more favourable for them to climb at steeper inclines. Climbing simulates the effect of oncoming cells pushing up and under cells causing them to rise upwards to a new layer. It was found that cells need to climb at a fairly steep angle (though only for relatively short distances) with a typical incline angle being 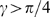 rad since a cell is being pushed upwards by another cell so the height it rises must be sufficient to allow the opposing cell to move into its space.

Sozinova *et al.* [18] used a three-dimensional lattice gas cellular automata model to study rippling formation. Cells were oriented in one of six directions on a hexagonal grid, which introduces spatial inaccuracy. This limits the direction cells can move in and any orbiting patterns of cells may be an artefact of this; any alteration in direction is a turn of 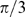 rad so cells can move through tight arcs. The rigid cell body also means that the cell must alter its course dramatically. In reality, the partial flexibility of the cell means it does not have to completely alter its course to avoid an obstacle; it can bend slightly to align itself alongside the object and move around it. O'Connor and Zusman [11] showed that cells cluster in small aligned patches within a fruit and move together. A hexagonal lattice model does not allow for this; cells maintain alignment only if they never change direction, otherwise they alter course by 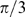 rad and spatial alignment is lost almost immediately.

The models presented here show that it is possible for fruiting bodies to develop without artificial induction. Cell density and an upward pushing force seem to be sufficient to instigate formation. Importantly, the EPS surrounding cells must exert an adhesive force, binding cells together. Without this force, cells are too unconstrained and move away from the aggregate. Each layer acts almost independently. Cells from one layer have a much reduced effect on cells in another layer than cells in the same layer. Experiments where all terms in the Hamiltonian were dependent on a local three-dimensional neighbourhood showed that cells cannot move freely due to feeling the effects of cells moving in all directions around them.

The fruiting models offer an explanation of the initial formation of fruiting bodies as a consequence of cell physics and a low reversal frequency, suggesting that fruiting bodies form can form spontaneously without the need for an artificial aggregation centre to start the process. The fruiting model has also shown that observed transitory fruiting body developments before a stable fruiting body forms can be explained as a consequence of net cell influx. Sporulation appears to be important for stable fruiting body formation. A large aggregate of non-motile cells provides a base around which the motile cells can move to form the fruiting body. Motile cells are still driven upwards at the base of the aggregate where streams collide and they force the spores to move upwards as well. This offers a potential mechanism for allowing myxobacterial cells to form sporangiole on stalks without extensive behaviour modifications. Although the model incorporates sporulation, it is still not clear how cells choose to sporulate since only a percentage of the fruiting body do so. The fruiting model approximates this behaviour by only allowing a percentage of the cells to sporulate. Future experimental work will hopefully provide more information on the sporulating process which can be incorporated into the models.

The models presented here offer potential mechanisms *M. xanthus* could use to organise streaming, aggregation and fruiting body formation. Importantly, by deriving the fruiting models from an existing model of rippling, we have shown that a single model based on the observable, physical characteristics of myxobacteria can explain multiple spatial phenotypes and may shed more light on how myxobacteria is able to exhibit multiple different behaviours during its life-cycle.

## Supporting Information

Figure S1 Fruiting body formation with a finite number of cells. 1600 cells were divided into four opposing streams. A fruiting body starts to form after 100 min. There are not enough cells to sustain fruiting body growth beyond a few layers and cells dissociate after 400 min. Plots are a two-dimensional (xy-plane) top down view of a three-dimensional environment. Cells are coloured by height; the darker the grey, the higher the cell has climbed.
(1.22 MB TIF)

Figure S2 Converging stream formations. Fruiting body simulations begin with streams of cells converging to form an aggregate. To maintain cell density within the fruit, the initial stream formations are augmented with four cell influx regions, one at each boundary of the simulation (in the xy-plane). Cells are created at the influx regions and allowed to move into the simulation volume. (A) Diagram view of simulation. (B) Snapshot of a simulation after 20 time steps showing the creation of cells. (C) Snapshot of the same simulation after 50 time steps showing the formation of streams of cells converging towards the centre of the simulation. Cells move in the direction of their head (red segments) from their tail (blue segments).
(0.80 MB TIF)

Figure S3 Simulation of the effects of adhesion on stream formation. As adhesion becomes stronger cells cannot break apart and remain together in tighter clusters until the slime effectively becomes so viscous, cells cannot move. The head, tail and body of each cell are coloured red, blue and black respectively. Plots are a two-dimensional (xy-plane) top down view of a three-dimensional environment. (A) ϕ = 0. (B) ϕ = 10. (C) ϕ = 40. (D) ϕ = 50.
(0.86 MB TIF)

Figure S4 Fruiting body simulation using a finite number of cells. 1600 cells in two (perpendicular) opposing sets of streams. In the centre, cells move over others, forming the base of a stalk. After 300 time steps the stalk begins to disassociate.
(2.16 MB TIF)

Video S1 Video of simulated fruiting body aggregation - Simulation is shown active at the 20 min, 500 min, 1000 min and 1500 min time points to illustrate the formation of the fruiting body.
(2.26 MB MOV)
