# Supplementary figures and images for: Spatial Simulations of Myxobacterial Development
Source: PLoS Comput Biol. 2010 Feb 26;6(2):e1000686. doi: 10.1371/journal.pcbi.1000686 (PMC2829040; doi:10.1371/journal.pcbi.1000686)

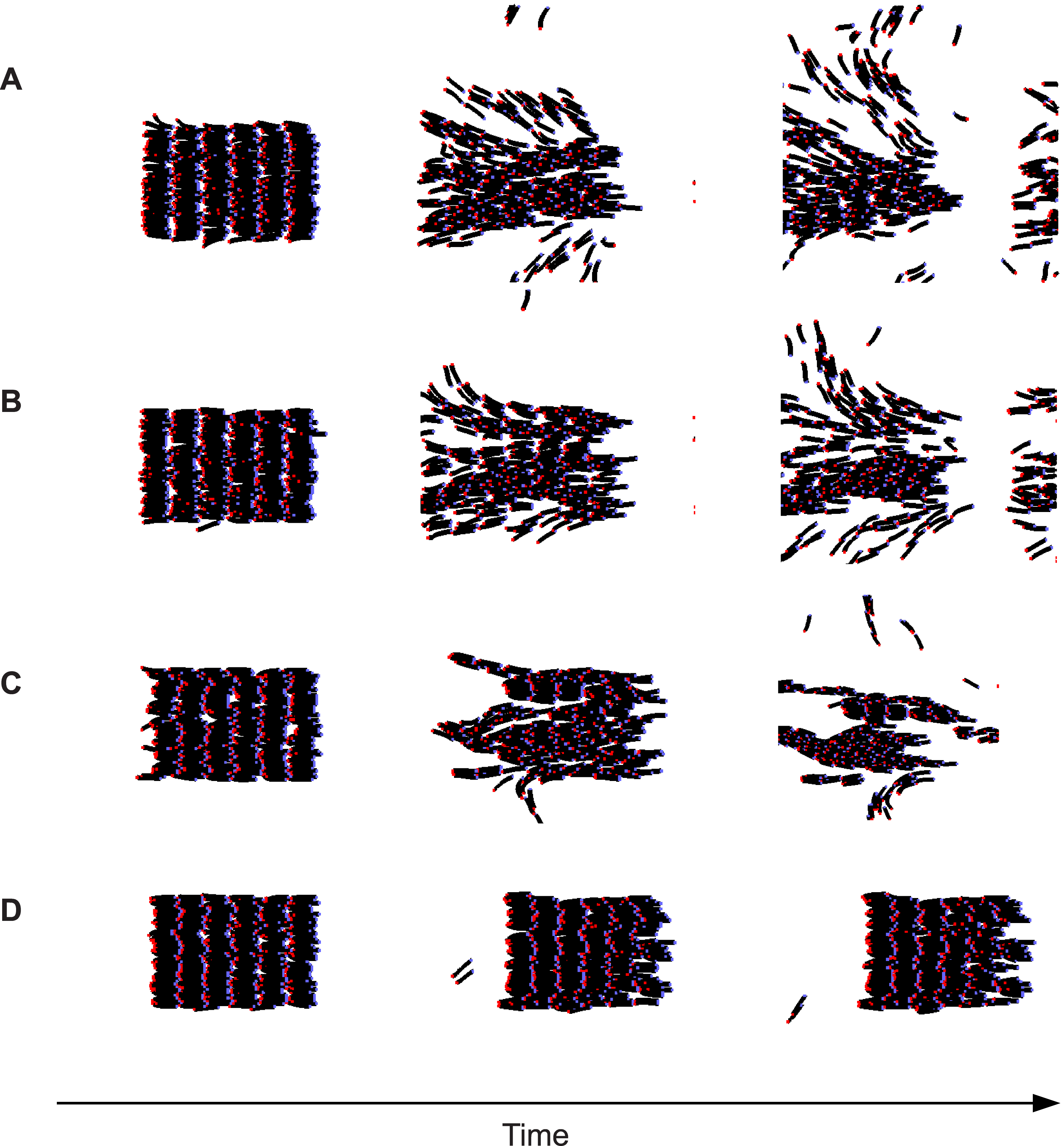

Supplement: Figure S1 — Fruiting body formation with a finite number of cells. 1600 cells were divided into four opposing streams. A fruiting body starts to form after 100 min. There are not enough cells to sustain fruiting body growth beyond a few layers and cells dissociate after 400 min. Plots are a two-dimensional (xy-plane) top down view of a three-dimensional environment. Cells are coloured by height; the darker the grey, the higher the cell has climbed. (1.22 MB TIF) [file pcbi.1000686.s001.tif]

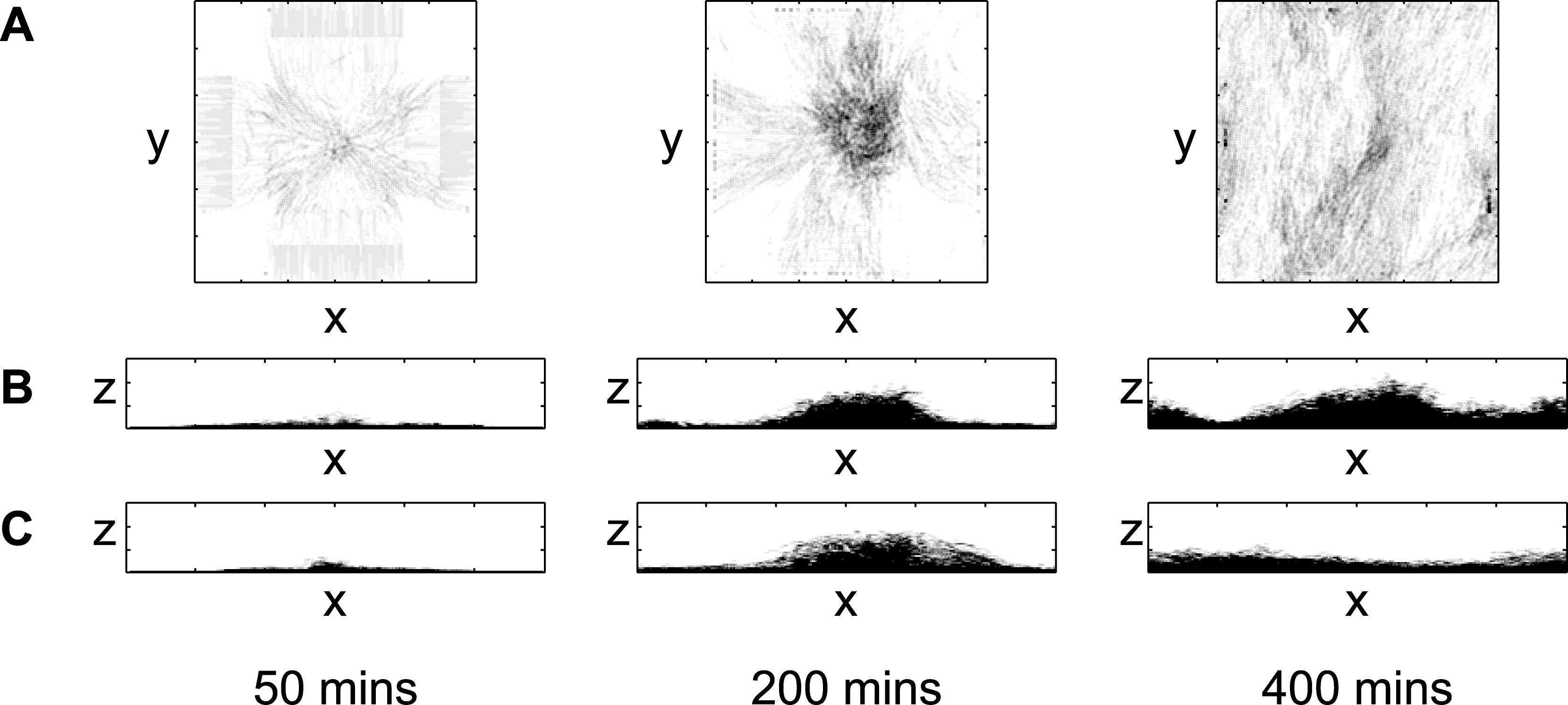

Supplement: Figure S2 — Converging stream formations. Fruiting body simulations begin with streams of cells converging to form an aggregate. To maintain cell density within the fruit, the initial stream formations are augmented with four cell influx regions, one at each boundary of the simulation (in the xy-plane). Cells are created at the influx regions and allowed to move into the simulation volume. (A) Diagram view of simulation. (B) Snapshot of a simulation after 20 time steps showing the creation of cells. (C) Snapshot of the same simulation after 50 time steps showing the formation of streams of cells converging towards the centre of the simulation. Cells move in the direction of their head (red segments) from their tail (blue segments). (0.80 MB TIF) [file pcbi.1000686.s002.tif]

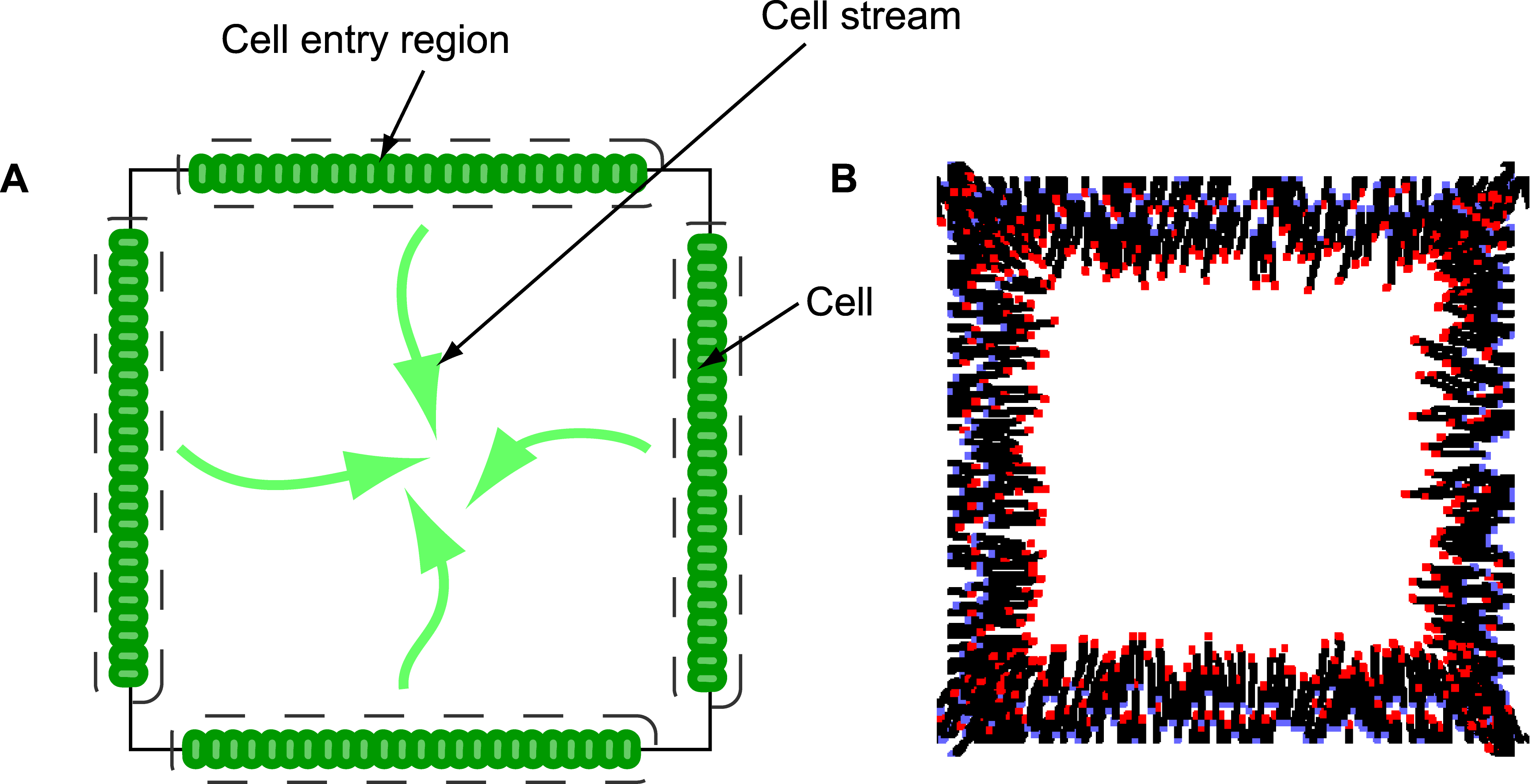

Supplement: Figure S3 — Simulation of the effects of adhesion on stream formation. As adhesion becomes stronger cells cannot break apart and remain together in tighter clusters until the slime effectively becomes so viscous, cells cannot move. The head, tail and body of each cell are coloured red, blue and black respectively. Plots are a two-dimensional (xy-plane) top down view of a three-dimensional environment. (A) ϕ = 0. (B) ϕ = 10. (C) ϕ = 40. (D) ϕ = 50. (0.86 MB TIF) [file pcbi.1000686.s003.tif]

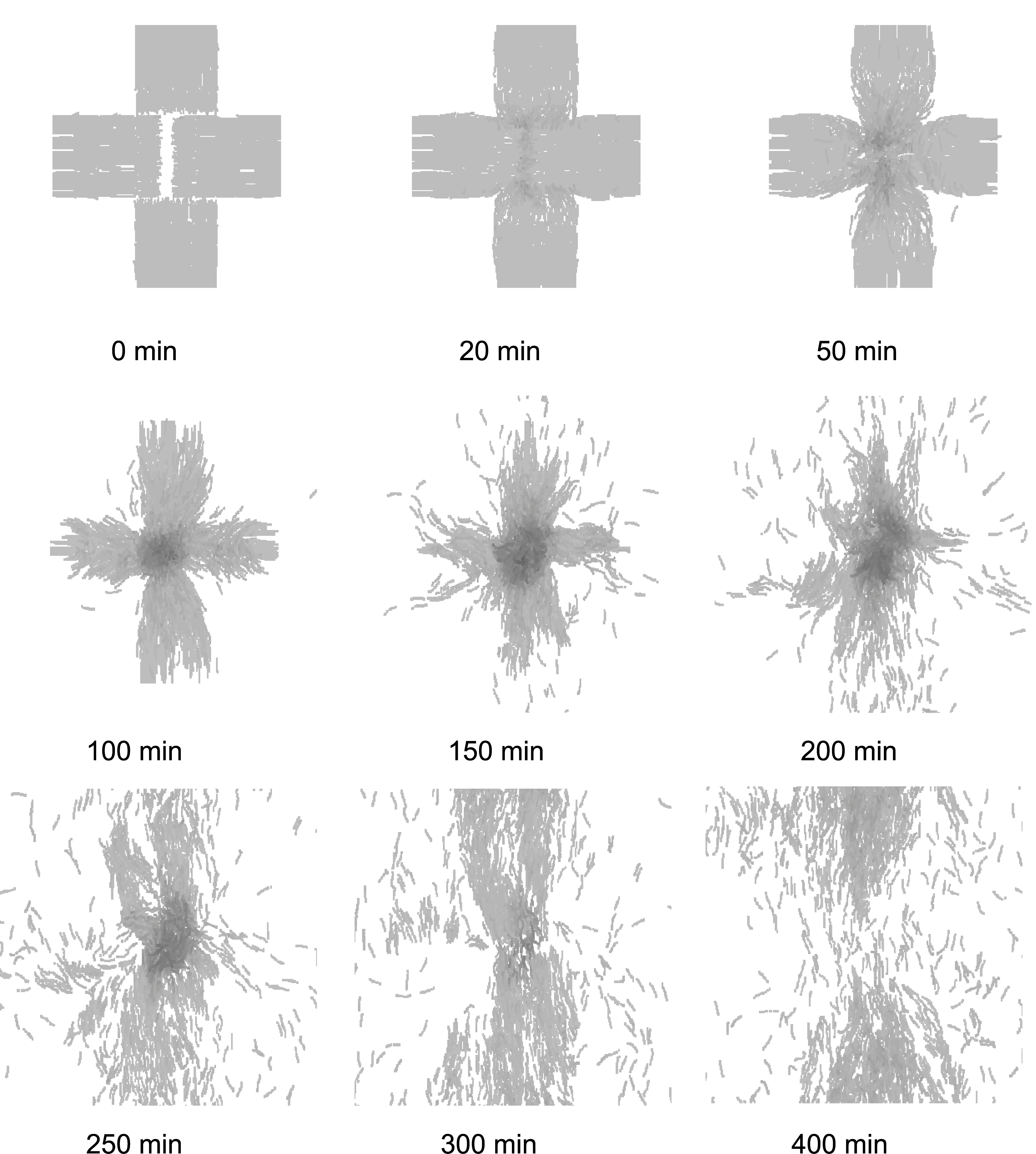

Supplement: Figure S4 — Fruiting body simulation using a finite number of cells. 1600 cells in two (perpendicular) opposing sets of streams. In the centre, cells move over others, forming the base of a stalk. After 300 time steps the stalk begins to disassociate. (2.16 MB TIF) [file pcbi.1000686.s004.tif]
